# Host-derived MMP-13 exhibits a protective role in lung metastasis of melanoma cells by local endostatin production

**DOI:** 10.1038/bjc.2011.431

**Published:** 2011-10-20

**Authors:** H Fukuda, S Mochizuki, H Abe, H J Okano, C Hara-Miyauchi, H Okano, N Yamaguchi, M Nakayama, J D'Armiento, Y Okada

**Affiliations:** 1 Department of Pathology, School of Medicine, Keio University, 35 Shinanomachi, Shinjuku-ku, Tokyo 160-0016, Japan; 2 Department of General Thoracic Surgery, Saitama Medical Center, Kawagoe, Saitama 350-8550, Japan; 3 Department of Physiology, School of Medicine, Keio University, 35 Shinanomachi, Shinjuku-ku, Tokyo 160-0016, Japan; 4 Department of Cell Biology and Morphology, Akita University Graduate School of Medicine and Faculty of Medicine, Akita 010-8543, Japan; 5 Division of Molecular Medicine, Department of Medicine, Columbia University College of Physicians and Surgeons, New York, NY 10032, USA

**Keywords:** matrix metalloproteinase-13, metastasis, melanoma, endostatin, SDF-1*α*, migration

## Abstract

**Background:**

Although matrix metalloproteinases (MMPs) are implicated in tumourigenesis and cancer progression, the role of MMP-13 in melanoma cell metastases is poorly understood.

**Methods:**

Lung metastases of mouse melanoma B16BL6 cells were analysed in MMP-13 knockout (KO) and wild-type (WT) mice after intravenous injection. The mRNA and protein expression of MMP-13 in lung tissues was analysed by RT–PCR, real-time PCR, immunoblotting and immunohistochemistry. The expression of SDF-1*α*, CXCR4 and endostatin, and effects of endostatin to cultured melanoma cells and lung metastases were also studied.

**Results:**

Lung metastases of B16BL6 cells were significantly higher by 2.5–5.7-fold in MMP-13 KO mice than in WT mice. The expression of MMP-13 in WT mouse lung tissue was stimulated on day 1 after intravenous injection of the melanoma cells and MMP-13 was immunolocalised to vascular endothelial cells in the lungs. Endostatin formation, but not degradation of SDF-1*α*, in the lung tissue was associated with reduced lung metastasis in WT mice. Endostatin significantly inhibited migration of B16BL6 cells in monolayer wounding assay and remarkably suppressed Matrigel invasion and transendothelial invasion of the cells. In addition, lung metastases of melanoma cells in MMP-13 KO mice were reduced by intraperitoneal administration of endostatin.

**Conclusion:**

Our results suggest that MMP-13 is overproduced by endothelial cells in the lungs with melanoma cells and has a protective role in lung metastasis by local generation of endostatin.

The lung is one of the most common sites of cancer cell metastases. Most malignant tumours (both carcinomas and sarcomas) arising anywhere in the body preferentially metastatise to the lungs with the exception of carcinomas of the digestive organs such as the gastrointestinal tract, which exhibits metastases mainly in the liver. According to the ‘anatomical theory’ for metastases, this may be explained by the fact that whole blood passes through the lungs and the cancer cells in the blood form tumour emboli within capillaries (Chambers *et al*, 2002). On the other hand, the ‘seed and soil theory’ suggests that the lung tissue microenvironment contains many molecules that modulate lung-specific metastases of cancer cells, which include growth factors, cytokines, chemokines, cell adhesion molecules, proteinases and transcriptional factors (Chambers *et al*, 2002; Minn *et al*, 2005; Fokas *et al*, 2007). However, the molecular mechanism by which lung metastases are controlled remains elusive.

The matrix metalloproteinase (MMP) gene family contains 23 members in humans and some MMPs are considered to have key roles in invasion and metastases of various cancers by degrading the extracellular matrix (ECM) and/or non-ECM molecules such as cytokines, chemokines and growth factors (Chambers *et al*, 2002; Shiomi and Okada, 2003; Deryugina and Quigley, 2006). In the murine system, MMP-1 has a very restricted expression and thus MMP-13 most likely represents the main collagenase (Egeblad and Werb, 2002; Shiomi *et al*, 2010). Although MMP-13 is overexpressed in a variety of malignant tumour tissues (Pendas *et al*, 2000), the distribution of MMP-13 within tumours, that is, the expression by tumour cells and/or stromal cells, appears to be dependent on the tumour type. MMP-13 is expressed predominantly by tumour cells in squamous cell carcinomas of the head, neck and vulva (Johansson *et al*, 1997, 1999; Jasani *et al*, 1998) and in cutaneous basal cell carcinomas (Airola *et al*, 1997). However, in breast carcinomas (Uria *et al*, 1997; Pendas *et al*, 2000) and melanomas (Zigrino *et al*, 2009), MMP-13 is reportedly expressed almost exclusively by stromal fibroblasts and/or endothelial cells within or near the tumours. The selective expression of MMP-13 by stromal fibroblasts neighbouring tumour islands is also reported in skin tumours developed in the human papillomavirus type 8 transgenic mouse (Akgul *et al*, 2006). Zigrino *et al* (2009) have recently shown that tumour growth and organ metastases of mouse melanoma B16F1 cells, which have no expression of MMP-13, are reduced when they are inoculated in the dermis in MMP-13 knockout (KO) mice. This study suggests that stromal cell-derived MMP-13 promotes melanoma cell growth in the skin and organ metastases from the primary cutaneous melanoma. However, metastasis is a multistep process which comes from cells escaping from the primary tumour, invading the surrounding tissues, entering the vasculature, reaching secondary sites, extravasating from the vessels and then establishing metastatic foci (Chambers *et al*, 2002; Deryugina and Quigley, 2006). Although multiple studies have demonstrated the critical function of MMPs including MMP-13 in tumour cell invasion of surrounding tissues (Liotta and Kohn, 2001; Deryugina and Quigley, 2006), little or no information is thus far available for the role of MMP-13 in tumour cell extravasation.

In the present study, we aimed to examine the role of host-derived MMP-13 in the development of lung metastases of mouse B16BL6 melanoma cells by focusing on the extravasation step after the intravenous injection of cells into the MMP-13 KO and wild-type (WT) mice. To the best of our knowledge, our study provides the first evidence that MMP-13 overproduced by endothelial cells in the lungs with melanoma cells has a protective role in lung metastasis by reducing the extravasation through the local generation of endostatin.

## Materials and methods

### Animals

MMP-13 KO mice on a 129/Sv genetic background were generated by microinjection of ES cells into C57BL/6J blastocytes as described previously (Takaishi *et al*, 2008). The mice were backcrossed at least 10 times into the C57BL/6J background, and genotyping of animals was performed by PCR of DNA obtained from tail biopsies (Hattori *et al*, 2009). MMP-13 KO mice exhibited a normal lifespan with sufficient fertility and did not show a gross phenotype after maturation, although they exhibited transient growth retardation due to defects in the growth plate during development (Takaishi *et al*, 2008; Hattori *et al*, 2009). Matched control littermates of MMP-13 KO mice were used as WT mice for experiments.

### Macroscopic and histological analyses of lung metastases

Lung metastases were generated through the injection of mouse B16BL6 melanoma cells (RIKEN BioResource Center, Tsukuba, Japan) into the tail veins of MMP-13 KO and WT mice. To initially evaluate the lung metastases, we carried out pilot studies by the injection of varying numbers of melanoma cells (5 × 10^3^, 5 × 10^4^ and 5 × 10^5^ cells per mouse) into minimum number of the mice (*n*=5) according to the guideline described by Workman *et al* (2010). All of the MMP-13 KO and WT mice received 5 × 10^5^ cells per mouse died by 3 weeks, whereas the mice injected with 5 × 10^3^ cells per mouse exhibited only a small number of metastases in the lungs at 3 weeks: 0.5±1.0 and 4.2±2.5 (mean±s.d.) metastatic foci on the lung surface of WT and MMP-13 KO mice, respectively (*n*=5 per group; data not shown). Therefore, we injected 5 × 10^4^ cells per mouse into the tail veins of the mice in the present study.

Mice were killed at 3 weeks after the injection by intraperitoneal administration of overdosed Nembutal (Dainippon Sumitomo Pharma Co., Ltd, Osaka, Japan), and the lung and heart were removed *en bloc*. Lung metastases were macroscopically counted and lung weight was measured. Then, the lungs were fixed by intratracheal injection of 4% buffered formalin and embedded in paraffin. Sections that included the trachea were prepared, and the number and area of metastatic nodules (% tumour area of the whole lung) were measured by quantitative morphometric planimetry (Matsumura *et al*, 2005). All procedures were performed according to the guidelines for the Care and Use of Laboratory Animals of School of Medicine, Keio University and those by Workman *et al* (2010).

### Establishment of B16BL6 melanoma cells expressing Venus and luciferase and evaluation of lung metastases

The lentiviral expression vectors containing the fusion gene of Venus (a variant of green fluorescent protein) and luciferase (Okada *et al*, 2005) were transfected into B16BL6 melanoma cells using Lipofectamin 2000 (Invitrogen, Carlsbad, CA, USA). Two days after transfection to 293FT cells, the lentiviral supernatants were collected and filtered using a Millex filter (0.45 *μ*m; Millipore, Bedford, MA, USA). B16BL6 melanoma cells were infected with the virus-containing media for 48 h, continuously propagated, and then dissociated into single cells. The Venus-positive cells (B16BL6^Venus−Luc^ cells) were sorted by a MoFlo flow cytometer (Cytomation, Fort Collins, CO, USA) and maintained in RPMI-1640 with 10% fetal bovine serum (FBS).

B16BL6^Venus−Luc^ cells were injected into the tail veins of MMP-13 KO and WT mice (5 × 10^4^ cells per mouse). Lung metastases were monitored by bioluminescence imaging using the IVIS-100 camera system (Xenogen, Alameda, CA, USA) to detect luciferase expression according to the manufacturer's instructions. Mice received an intraperitoneal injection of D-luciferin (150 mg kg^−1^; Promega, Madison, WI, USA) and 15 min later, photons from the whole body of the animal were counted. Data were analysed by using LIVING IMAGE 3.0 software (Xenogen).

### Reverse transcription-PCR for MMP-13, SDF-1*α*, CXCR4 and *β*-actin

Total RNA was extracted from the lungs of MMP-13 KO and WT mice, and cDNA was prepared from 1 *μ*g of total RNA with ReverTra Ace (TOYOBO Co. Ltd., Osaka, Japan; Mitsui *et al*, 2006). Reaction products were subjected to reverse transcription-PCR (RT–PCR) analysis at 30 cycles for the expression of MMP-13, SDF-1*α* and CXCR4 and at 25 cycles for *β*-actin. Sequences of the primers were as follows: for MMP-13 5′-GGTCCCAAACGAACTTAACTTACA-3′ (forward), 5′-CCTTGAACGTCATCATCAGGAAGC-3′ (reverse); for SDF-1*α* 5′-TCTCGGTCCACCTCGGTGTCC-3′ (forward), 5′-GCTTTCTCCAGGTACAGGTACTCTTGGA-3′ (reverse); for CXCR-4 5′-GGTCTGGAGACTATGACTCC-3′ (forward), 5′-CACAGATGTACCTGTCATCC-3′ (reverse); for *β*-actin 5′-TTCTACAATGAGCTGCGTGTGGC-3′ (forward), 5′-CTCATAGCTCTTCTCCAGGGAGGA-3′ (reverse). The nucleotide sequences of the amplified fragments were confirmed by cycle sequencing using a DYEnamic ET dye terminator cycle sequencing kit (MegaBACE; Amersham Pharmacia Biotech, Schenectady, NY, USA) and MegaBACE 1000 DNA sequencer (Amersham Pharmacia Biotech) (Mitsui *et al*, 2006; Hattori *et al*, 2009). For quantitative analysis of MMP-13 expression in the lung tissues of WT and MMP-13 KO mice before and 1 day after B16BL6 melanoma cell injection, the cDNA was amplified in a SYBR Green real-time PCR assay (Invitrogen) according to the manufacturer's protocols. The relative quantification values of MMP-13 were normalised by those of an endogenous control, *β*-actin. Nucleotide sequences of the primers were 5′-GATGGCACTGCTGACATCAT-3′ (forward) and 5′-CCCACCATAGTTTGGTCCAG-3′ (reverse) for MMP-13; 5′-AGAGGGAAATCGTGCGTGAC-3′ (forward) and 5′-TAGTGATGACCTGGCCGT-3′ (reverse) for *β*-actin.

### Histology and immunohistochemistry for MMP-13

Lung tissues were fixed with 4% buffered formalin, and frozen sections (6 *μ*m) were stained with haematoxylin and eosin. For immunohistochemistry of MMP-13, sections were treated with rabbit anti-MMP-13 polyclonal antibody (1 : 100 dilution; H-230; Santa Cruz Biotechnology, Santa Cruz, CA, USA) after blocking for endogenous peroxidase and non-specific binding (Hattori *et al*, 2009). Then, they were incubated with peroxidase-conjugated secondary antibody (1 : 200 dilution; Envision; Dako, Glostrup, Denmark). The control sections were treated with non-immune rabbit IgG replacing the first antibody.

### Immunoblotting for MMP-13, endostatin and *β*-actin

Lung tissues were obtained from MMP-13 KO and WT mice after the intravenous injection of B16BL6 cells. Homogenate supernatants (50 *μ*g per lane for MMP-13 and *β*-actin and 30 *μ*g per lane for endostatin) were separated by sodium dodecyl sulphate-polyacrylamide gel electrophoresis under reducing conditions and the proteins resolved on the gels were transferred onto PVDF membranes. The membranes were blotted with anti-MMP-13 antibody (1 : 1000 dilution; H-230; Santa Cruz Biotechnology), anti-endostatin antibody (1 : 500 dilution; AF570; R&D Systems Inc., Minneapolis, MN, USA) or anti-*β*-actin antibody (1 : 1000 dilution; AC-74; Sigma-Aldrich, St Louis, MO, USA), and densitometric analysis was carried out according to our previous methods (Matsumura *et al*, 2005; Hattori *et al*, 2009).

### Enzyme-linked immunosorbent assay for SDF-1*α*

The concentration of SDF-1*α* in the lung homogenate and serum samples was assayed by the Quantikine mouse CXCL12/SDF-1*α* ELISA kit (MCX120; R&D Systems Inc.) (Supplementary Methods).

### *In vitro* B16BL6 melanoma cell migration assay

B16BL6 cells were grown to confluence on 6-well plates (BD Biosciences, Bedford, MA, USA) in RPMI-1640 containing 10% FBS, 1% antibiotic solution, and then scratch wounded with a blue pipette tip according to the previous methods (Liang *et al*, 2007). The cells were allowed to migrate in the presence and absence of endostatin (Yamaguchi *et al*, 1999) in the medium containing 5 mM hydroxyurea, an inhibitor of cell proliferation. The marked areas of the wound were photographed, and cell migration areas were determined using Image J software (NIH, Bethesda, MD, USA).

### Matrigel invasion and transendothelial invasion assays

The effects of endostatin on the invasion of B16BL6 melanoma cells were analysed in 24-well Matrigel invasion chambers (BD Biosciences). The Matrigel matrix insert (8 *μ*m pores) was used to separate upper and lower compartments. An aliquot (200 *μ*l) of a melanoma cell suspension (1 × 10^6^ cells per ml) in RPMI-1640 with 0.1% bovine serum albumin was added to the upper compartment of the chamber in the presence and absence of endostatin. The lower compartment of the chamber was charged with 10% FBS. The chamber was incubated at 37 °C for 8 h in a humidified incubator containing 95% air and 5% CO_2_. The net number of cells that invaded through the 8-*μ*m pores was determined in three random high-power fields for each filter, which was stained with Diff-Quick dye (Matsumura *et al*, 2005). For transendothelial invasion assay, human umbilical vein endothelial cells (LONZA, Walkersville, MD, USA) were cultured on 24-well Matrigel invasion chambers for 2 days, and then melanoma cells (2 × 10^5^ cells) were added to the chambers in the presence and absence of endostatin (Yamaguchi *et al*, 1999). They were cultured for 20 h and the number of the invading cells was counted as described above. These assays were performed in triplicates.

### Administration of endostatin into MMP-13 KO mice after intravenous injection of melanoma cells

B16BL6^Venus−Luc^ cells were injected into the tail veins of MMP-13 KO and WT mice (5 × 10^4^ cells per mouse) (*n*=8 for MMP-13 KO mice and *n*=4 for WT mice). MMP-13 KO mice were randomly classified into two groups (*n*=4 per group) and received intraperitoneal injections of endostatin in phosphate-buffered saline (PBS; 2 mg kg^−1^ per day) or PBS alone on days 1–4 after melanoma cell injection by modification of the previous methods (Dhanabal *et al*, 1999; Sim *et al*, 1999). At 2 weeks after the melanoma cell injection, lung metastases in the mice of each group were measured by IVIS-100 camera system as described above.

### Statistical analyses

Statistical differences were determined using the Mann–Whitney *U*-test or Student's *t*-test. *P*-values <0.05 were considered significant.

## Results

### Enhanced lung metastases of B16BL6 melanoma cells in MMP-13 KO mice

**Figure 1:**
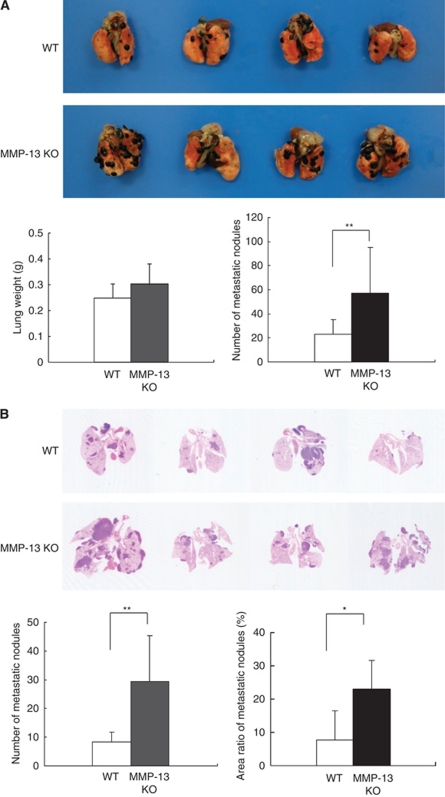
Lung metastases in WT and MMP-13 KO mice after intravenous injection of B16BL6 melanoma cells. (**A**) Representative macroscopic views of lung metastases and evaluation of lung weight and number of metastatic foci on the lung surface at 3 weeks after the injection of 5 × 10^4^ melanoma cells into WT and MMP-13 KO mice (*n*=15 per group). Note that metastatic foci are seen as black nodules on the surface of the lungs. Metastatic foci were counted by stereoscopic microscope. Bars, mean±s.d. ^**^P<0.01. (**B**) Representative microscopic views of the lung sections and evaluation of number of metastatic foci and area ratio of metastatic nodules in WT and MMP-13 KO mice (*n*=7 per group). Paraffin sections stained with haematoxylin and eosin show that metastatic foci are seen as haematoxylin-positive blue-coloured lesions. The number per lung was counted, and area ratio of metastatic foci to whole lung was calculated using Image J software. Bars, mean±s.d. ^*^P<0.05; ^**^P<0.01.

The effects of host-derived MMP-13 on lung metastasis were examined by comparing the metastases in the lungs 3 weeks after injecting B16BL6 mouse melanoma cells into tail veins of MMP-13 KO and WT mice. Although the lung weight was not significantly different between MMP-13 KO and WT mice, the number of metastatic foci on the lung surface was significantly higher by a factor of 2.5 in the MMP-13 KO mice compared with WT mice (57±38 *vs* 23±11; mean±s.d.; *P*<0.01; Figure 1A). Microscopic examination of the sections of the lungs showed that the number of metastatic nodules was 3.6-fold significantly higher in MMP-13 KO mice than in WT mice (29.5±15.8 *vs* 8.2±3.6; *P*<0.01; Figure 1B). Similarly, the nodular area was 2.9-fold significantly higher in MMP-13 KO mice than in WT mice (22.7±8.6% *vs* 7.7±8.7% *P*<0.05; Figure 1B).

**Figure 2:**
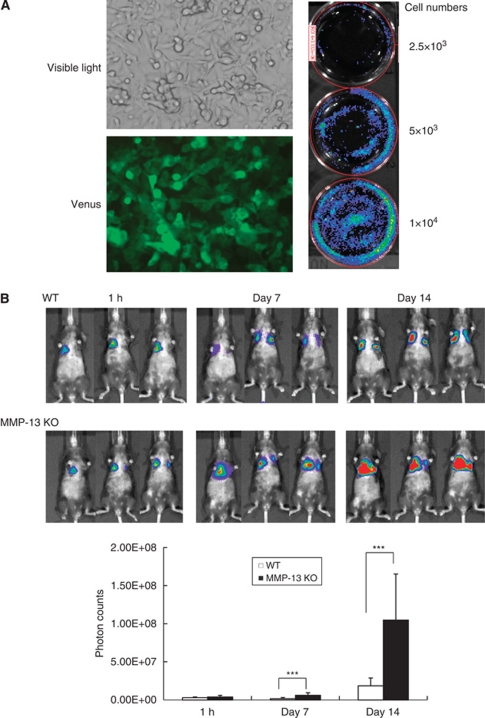
Establishment of B16BL6^Venus−Luc^ melanoma cells and monitoring of lung metastases after the intravenous injection into WT and MMP-13 KO mice. (**A**) Establishment of B16BL6^Venus−Luc^ melanoma cells. The cells show positive staining under fluorescence microscopy for Venus, and photon counts are dependent on the cell number after addition of luciferin to culture media of the cells. (**B**) Time-course changes of lung metastases after intravenous injection of B16BL6^Venus−Luc^ cells. Photon counts of whole lungs were counted at 1 h, 7 days and 14 days after intravenous injection of 5 × 10^4^ cells into WT and MMP-13 KO mice (*n*=5 per group) by using LIVING IMAGE 3.0 software. Bars, mean±s.d. ^***^P<0.001.

To further confirm the data and monitor the lung metastases, we developed an experimental model using bioluminescence imaging. As shown in Figure 2A, B16BL6^Venus–Luc^ melanoma cells stably transfected with vectors containing the Venus–luciferase fusion gene exhibited positive staining under fluorescence microscopy for Venus (91.1±1.9%). Upon addition of luciferin to culture media of the cells, photon counts were dependent on the cell numbers (Figure 2A), showing a direct correlation between them (*r*=0.98; data not shown). The B16BL6^Venus−Luc^ cells were injected via tail veins of MMP-13 KO and WT mice, and photon counts of the mice were measured chronologically by bioluminescence imaging after intra-abdominal administration of luciferin. Figure 2B shows that although photon counts are similar in MMP-13 KO and WT mice at 1 h after melanoma cell injection, they significantly increase by 3.8-fold and 5.7-fold on days 7 and 14, respectively. The above-described data utilising B16BL6 melanoma cells suggest that MMP-13 has a protective effect on the formation of lung metastasis.

### Expression of MMP-13 in lung tissues after intravenous injection of melanoma cells

**Figure 3:**
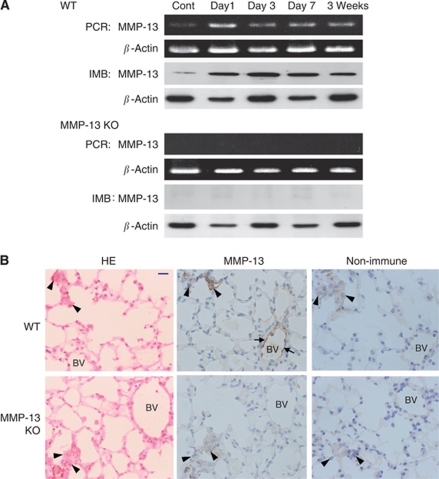
The expression and immunolocalisation of MMP-13 in the lung tissues of WT and MMP-13 KO mice after intravenous injection of B16BL6 melanoma cells. (**A**) The mRNA and protein expression of MMP-13 in the lungs of WT and MMP-13 KO mice. Mice were received intravenous injection of B16BL6 cells and lung tissues obtained on days 0 (Cont), 1, 3, 7 and 21 (3 weeks) after the injection were subjected to RT–PCR (PCR) and immunoblotting (IMB) for MMP-13 as described in Materials and Methods. *β*-Actin, a loading control. (**B**) Histological examination of metastatic foci and immunohistochemical localisation of MMP-13 in the lungs of WT and MMP-13 KO mice on day 3 after the injection. Arrows indicate immunoreactive endothelial cells and arrowheads show melanoma cells that can be detected by large nuclei and/or melanin granules within the cytoplasm. Non-immune IgG instead of anti-MMP-13 antibody was used as for a control. Abbreviations: HE, haematoxylin and eosin staining; BV, blood vessel. Scale bar, 10 *μ*m.

The expression level of MMP-13 mRNA and protein in the lung tissues after intravenous injection of melanoma cells was monitored by RT–PCR and immunoblotting. As shown in Figure 3A, the mRNA level of MMP-13 in WT mice was low or negligible before the injection, but the level quickly increased and peaked on day 1 and continued to remain elevated until 3 weeks after the injection. Quantitative real-time PCR showed that the relative mRNA expression level of MMP-13 (MMP-13/*β*-actin ratio) is significantly 2.9-fold higher in the WT mouse group on day 1 after the injection (3.6±0.4) compared with the control group without the injection (1.2±0.4) (*P*<0.05), although negligible expression was obtained in MMP-13 KO mouse group (*n*=3 per group) (data not shown). Immunoblotting data provided similar results, confirming the RT–PCR and real-time PCR data (Figure 3A). In contrast, neither mRNA levels nor levels of MMP-13 protein were detectable in the lung tissue of MMP-13 KO mice even after intravenous injection of B16BL6 cells (Figure 3A). Indeed, B16BL6 cells in culture demonstrated no expression of MMP-13 by RT–PCR (data not shown). Immunohistochemistry using anti-MMP-13 antibody showed that MMP-13 is localised to the endothelial cells of the blood vessels in the lung tissues of WT mice, but not in MMP-13 KO mice (Figure 3B). No staining was observed in the tissue samples by immunostaining with non-immune IgG (Figure 3B).

### No significant relationship between SDF-1*α* expression and lung metastasis

Since the protective effect of MMP-13 on lung metastasis was associated with overproduction of MMP-13 by endothelial cells in the lung tissue at an early stage, we speculated that molecules implicated in the metastasis (either metastasis-promoting or anti-metastatic molecules) are modulated by MMP-13 activity in WT mice. Among the MMP-13 substrates that might influence metastasis (McQuibban *et al*, 2001; Heljasvaara *et al*, 2005), we first focused on the expression and degradation of SDF-1*α* (also called CXCL12), one of the chemokines that is known to promote lung metastasis through interaction with its receptor CXCR4 (Muller *et al*, 2001; Murakami *et al*, 2002). However, the mRNA expression of SDF-1*α* and its receptor CXCR4 in the lung tissue was similar on days 0, 1, 3 and 7 in WT and MMP-13 KO mice (Supplementary Figure 1). Protein levels of SDF-1*α* in the lung tissue and serum samples were monitored by the enzyme-linked immunosorbent assay (ELISA) system that detects intact and active SDF-1*α*, but there was no significant difference in the level of SDF-1*α* (Supplementary Figure 1). These indicate that the SDF-1*α*/CXCR4 axis is not related to the protective effect on lung metastasis by MMP-13 in WT mice.

### Increased production of endostatin in lung tissues of WT mice after intravenous injection of melanoma cells

**Figure 4:**
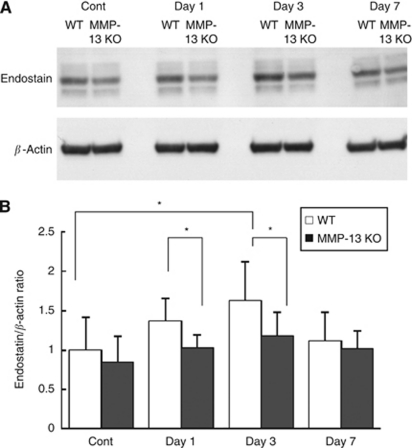
Immunoblotting analysis of endostatin in the lung tissues of WT and MMP-13 KO mice. (**A**) Representative data of immunoblotting of endostatin in the lung tissues of WT and MMP-13 KO mice. Mice were received intravenous injection of B16BL6 cells and lung tissues obtained on days 0 (Cont), 1, 3 and 7 after the injection were subjected to immunoblotting for endostatin as described in Materials and Methods. *β*-Actin, a loading control. (**B**) Time-course changes of endostatin/*β*-actin ratio on days 0 (Cont), 1, 3 and 7 (*n*=5 per group). Densitometric analysis was carried out using Image J software. Bars, mean±s.d. ^*^P<0.05.

We then examined the possible involvement of endostatin, which is a digestion product of type XVIII collagen by MMPs including MMP-13 (Heljasvaara *et al*, 2005) and is known to inhibit angiogenesis and migration of tumour cells (Yamaguchi *et al*, 1999; Kim *et al*, 2006). Immunoblotting analysis of endostatin in lung tissues showed that endostatin formation appears to be increased on days 1 and 3 in the WT mouse lungs compared with MMP-13 KO mouse lungs (Figure 4A). By densitometric analysis, the endostatin/*β*-actin ratio significantly increased 3 days after the melanoma cell injection into WT mice (*P*<0.05; Figure 4B). When the ratio was compared between WT and MMP-13 KO mice, it was significantly higher in the samples from WT mice on days 1 and 3 than in those from MMP-13 KO mice (*P*<0.05; Figure 4B).

### Inhibition of migration, invasion, transendothelial invasion and lung metastases of B16BL6 melanoma cells by endostatin

**Figure 5:**
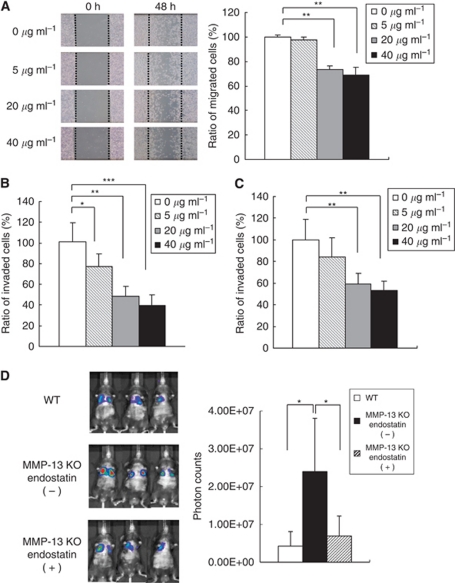
Inhibitory effects of endostatin on the migration, Matrigel invasion, transendothelial invasion and lung metastases of B16BL6 melanoma cells. (**A**) Effects of endostatin on the migration of the melanoma cells. B16BL6 melanoma cells in monolayer culture were wounded and migration activity was monitored at 24 and 48 h in the presence of endostatin (0, 5, 20 or 40 *μ*g ml^−1^) in culture medium containing 5 mM hydroxyurea. Migration areas of the melanoma cells were determined by measuring cell migration areas using Image J software. Results are expressed as percentage of migration area compared with control (0 *μ*g ml^−1^). Bars, mean±s.d. ^**^P<0.01. (**B**) Effects of endostatin on Matrigel invasion of B16BL6 melanoma cells. Melanoma cells were added to the upper compartment of Matrigel invasion chamber and number of the cells that invaded Matrigel matrix membrane was counted as described in Materials and Methods. Percentage of invaded cells compared with control (0 *μ*g ml^−1^) is shown. Bars, mean±s.d. ^*^P<0.05; ^**^P<0.01; ^***^P<0.001. (**C**) Effects of endostatin on transendothelial invasion of B16BL6 melanoma cells. Melanoma cells were added to the upper compartment of Matrigel invasion chamber that had been covered with an endothelial cell layer and number of the cells that invaded the endothelial cell layer and Matrigel membrane was counted. Percentage of invaded cells compared with control (0 *μ*g ml^−1^) is shown. Bars, mean±s.d. ^**^P<0.01. (**D**) Inhibition of lung metastases by restoration of endostatin in MMP-13 KO mice. Endostatin was intraperitoneally injected into MMP-13 KO mice on days 1, 2, 3 and 4 after intravenous injection of B16BL6^Venus−Luc^ cells and lung metastases were measured by bioluminescence imaging at 2 weeks after the injection. Photon counts were compared between WT mice and MMP-13 KO mice with or without endostatin administration. ^*^P<0.05.

The effect of endostatin on migration of melanoma cells was examined by a monolayer wounding assay. As shown in Figure 5A, migration after 48 h was significantly suppressed with endostatin at concentrations of 20 *μ*g ml^−1^ (73.7±2.7%) and 40 *μ*g ml^−1^ (68.8±6.1%) compared with the control (100±1.4%) (*P*<0.01). A Matrigel invasion assay also demonstrated that endostatin significantly reduced invasion of the melanoma cells in a dose-dependent manner: 77.0±12.7% (*P*<0.05), 48.8±9.5% (*P*<0.01) and 39.8±10.2% (*P*<0.001) compared with the control (100±18.6%) in the presence of 5, 20 and 40 *μ*g ml^−1^ endostatin, respectively (Figure 5B). The effect of endostatin on transendothelial invasion of melanoma cells was also evaluated by counting the cells that transmigrated through the endothelial cell layer on Matrigel. As shown in Figure 5C, transendothelial invasion was inhibited with endostatin at concentrations of 5, 20 and 40 *μ*g ml^−1^ to 83.8±13.0% (*P*=0.137), 59.9±7.1% (*P*<0.01) and 53.9±6.5% (*P*<0.01) compared with the control (100±18.7%), respectively. To further examine whether restoration of endostatin levels influences the enhanced lung metastases in MMP-13 KO mice, endostatin was intraperitoneally injected into the MMP-13 KO mice during the time period of extravasation, that is, on days 1, 2, 3 and 4 after the melanoma cell injection. Bioluminescence imaging demonstrated that administration of endostatin into MMP-13 KO mice significantly decreases the lung metastases to a similar level of those in WT mice (*P*<0.05; Figure 5D). All these data indicate that endostatin not only inhibits migration, Matrigel invasion and transendothelial invasion of melanoma cells *in vitro* but also reduces lung metastases *in vivo* probably through inhibition of extravasation.

## Discussion

To the best of our knowledge, we have provided the first evidence that lung metastases of B16BL6 melanoma cells are increased in MMP-13 KO mice after intravenous injection, indicating a protective role for MMP-13 in melanoma cell lung metastasis. We demonstrated the macroscopic and microscopic evidence of metastatic foci in the lungs of MMP-13 KO and WT mice and further demonstrated lesions by bioluminescence imaging of the mice after intravenous injection of B16BL6^Venus−Luc^ cells. Several lines of evidence have indicated that MMP species such as MMP-2, MMP-7, MMP-9 and MMP-14 (MT1-MMP) have key roles in invasion and metastases of cancer cells (Liotta and Kohn, 2001; Shiomi and Okada, 2003; Deryugina and Quigley, 2006). Thus, targeted deletion of these MMPs is generally expected to inhibit cancer cell proliferation, invasion and metastases. Indeed, tumourigenesis and metastases are reported to decrease in MMP-2 KO (Itoh *et al*, 1998), MMP-7 KO (Wilson *et al*, 1997) or MMP-9 KO mice (Itoh *et al*, 1999; Acuff *et al*, 2006a). However, several studies provided contradictory results, that is, a protective role of host MMPs in carcinogenesis, tumour growth or metastases by the experiments using MMP-3 KO (McCawley *et al*, 2004), MMP-7 KO (Acuff *et al*, 2006a), MMP-9 KO (Cornelius *et al*, 1998; Hamano *et al*, 2003) and MMP-12 KO mice (Acuff *et al*, 2006b). In addition, a completely protective role in cancer has been reported in MMP-8 KO mice, which exhibited enhanced carcinogenesis induced by chemical carcinogens (Balbin *et al*, 2003). Zigrino *et al* (2009) recently reported that tumour growth in the dermis and organ metastases of mouse melanoma B16F1 cells, a cell line similar to that used in the present study, are reduced in MMP-13 KO mice compared with WT mice. The findings appear to contradict the data in our current study. However, the experimental setting was different between the two studies: While we focused on the extravasation of the melanoma cells in the metastasis processes by investigating lung metastases after intravenous injection, their study analysed growth within the dermis and distant metastases from the primary tumour, which involve several steps such as invasion, intravasation and extravasation. Therefore, the data from the previous and present studies suggest that MMP-13 may have a dual role in metastases, that is, a promoting effect on the invasion and intravasation and a protective effect on the extravasation. In addition, these data suggest that extravasation of tumour cells from blood vessels is not a simple reverse process of intravasation.

Our study has demonstrated that the expression of MMP-13 within the lung tissue is dramatically upregulated on day 1 after the intravenous injection of melanoma cells in WT mice. B16BL6 cells do not express MMP-13 in culture or after intravenous injection into the MMP-13 KO mice. Thus, MMP-13 detected within the lung tissues of WT mice is derived from cells constituting the lung tissues. In fact, immunohistochemistry demonstrated that the endothelial cells of the lung blood vessels are, at least in part, responsible for the production of MMP-13. The mechanism by which MMP-13 is expressed by endothelial cells in the lungs is unclear in the present study. However, MMP-13 is known to be expressed by vascular endothelial cells in and around the tumour masses of B16F1 melanoma cells implanted in the mouse dermis (Zigrino *et al*, 2009) and in the branches of pulmonary arteries of mice exposed to cigarette smoke (Wright *et al*, 2007). Interleukin-1 (IL-1), tumour necrosis factor-*α* (TNF-*α*) and transforming growth factor-*β* are inducers and/or stimulators of MMP-13 (Pendas *et al*, 2000; Hattori *et al*, 2003; Leivonen *et al*, 2006). Since IL-1 and TNF-*α* are overexpressed in metastatic lung tumours (Margalit *et al*, 2003; Kim *et al*, 2009), it is possible to speculate that such cytokines stimulate vascular endothelial cells to upregulate MMP-13 expression.

MMP-13 is capable of digesting various ECM macromolecules including collagen types I, II, III, IV, IX, X, XIV and XVIII, proteoglycans, fibronectin, tenascin, laminin and perlecan and also non-ECM substrates such as SDF-1*α* (CXCL12) (McQuibban *et al*, 2001; Ala-aho and Kahari, 2005; Heljasvaara *et al*, 2005). Among these substrates, we first focused on SDF-1*α*, since SDF-1*α* is highly expressed by stromal cells in lung tissues and promotes lung metastases of CXCR4-expressing melanoma cells and breast carcinoma cells by stimulation of tumour cell adhesion to endothelial cells and tumour cell growth under stress (Muller *et al*, 2001; Murakami *et al*, 2002). Thus, once SDF-1*α* is inactivated by degradation with MMP-13 in the lung tissues, this could explain the decrease in lung metastases in the WT mouse group. However, there were no changes in the expression of SDF-1*α* and CXCR4 in the lung tissue or protein levels of SDF-1*α* in the lung and serum samples, indicating that this hypothesis is not applicable for our model. On the other hand, our study revealed the enhanced production of endostatin in the lung tissue of WT mice after intravenous injection of melanoma cells. Endostatin, a COOH-terminal fragment of type XVIII collagen, is formed by cleavage of the collagen by several MMPs including MMP-3, -7, -9, -13 and -20 (Heljasvaara *et al*, 2005) and other proteinases such as neutrophil elastase (Ferreras *et al*, 2000). In the present study, endostatin formation was closely linked with the expression of MMP-13 in the lung tissues of WT mice. However, since endostatin was observed in the lung tissues of MMP-13 KO mice as well as WT mice, proteinases other than just MMP-13 are involved in the basal production of endostatin in the lung tissues. Nevertheless, it is plausible that enhanced production of endostatin on days 1 and 3 after the melanoma cell injection is due to the proteolytic action of MMP-13.

Endostatin was originally isolated and characterised as a specific inhibitor of endothelial cell proliferation (O’Reilly *et al*, 1997; Yamaguchi *et al*, 1999). However, subsequent studies have shown that endostatin inhibits migration of tumour cells including B16BL6 melanoma cells and suppresses primary tumour and metastasis growth in experimental animal models (Dhanabal *et al*, 1999; Sim *et al*, 1999; Kim *et al*, 2006). In the current study, we confirmed the previous findings of inhibition of melanoma cell migration (Kim *et al*, 2006), but further demonstrated the inhibitor activity of endostatin to Matrigel invasion and transendothelial invasion. In addition, our data have demonstrated that restoration of the endostatin levels at the early stage of lung metastases (on days 1–4 after the intravenous melanoma cell injection) leads to a significant reduction of the metastases in MMP-13 KO mice. Since our metastasis model reflects mainly the ability of melanoma cells to extravasate from blood vessels, the reduced melanoma cell metastases in WT mice may be ascribed to decreased extravasation by endostatin formation with MMP-13 in the lung tissue. However, since MMP-13 has a broad spectrum of substrate specificity to both ECM and non-ECM molecules (McQuibban *et al*, 2001; Ala-aho and Kahari, 2005; Heljasvaara *et al*, 2005), it is likely that there are possible and unexpected roles of MMP-13 other than endostatin formation that have been undiscovered in the extravasation step of metastasis in the present model. Therefore, further studies such as overall proteomic analyses must be useful for better understanding the mechanisms of MMP-13-mediated protection of lung metastases besides endostatin formation.

In summary, the data in the current study have suggested that endostatin generated by the action of MMP-13 in the lung tissue is involved in reduced lung metastases of melanoma cells. It has been reported that a protective role of stromal cell-derived MMP-12 in lung metastatic tumour growth is due to inhibition of angiogenesis by angiostatin formation from plasminogen by MMP-12 (Acuff *et al*, 2006b). The protective role of MMP-9 in tumour growth has also been suggested through angiogenic mechanism by generating anti-angiogenic factors angiostatin (Cornelius *et al*, 1998) and tumstatin (Hamano *et al*, 2003). All of these data suggest that the generation of the anti-angiogenic factors in the tissue microenvironment is a common pathway for the protective MMPs to cancer cell growth and metastases, and may provide a clue to develop a new remedy against lung metastases by preventing from extravasation of tumour cells through modulation of tissue microenvironmental anti-angiogenic factors such as endostatin.
