# Supplementary material for: Host-derived MMP-13 exhibits a protective role in lung metastasis of melanoma cells by local endostatin production
Source: Br J Cancer. 2011 Oct 20;105(10):1615–24. doi: 10.1038/bjc.2011.431 (PMC3242531; doi:10.1038/bjc.2011.431)
Supplement: Supplementary Figure 1 [file bjc2011431x1.ppt]

## Slide 1
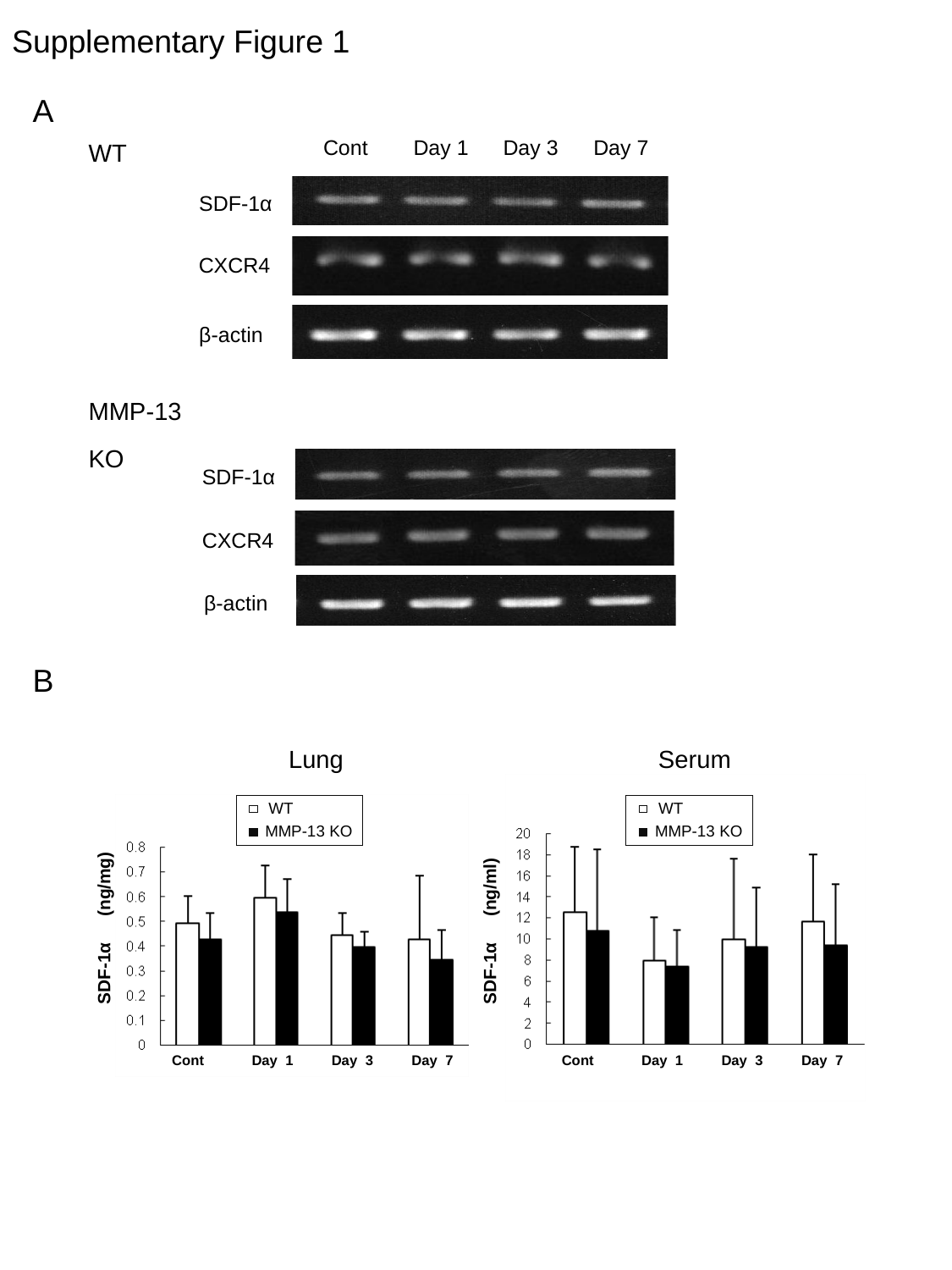

Supplementary Figure 1
A
WT
 Cont
 Day 1
 Day 3
 Day 7
SDF-1α
CXCR4
β-actin
MMP-13 KO
SDF-1α
CXCR4
β-actin
B
Lung
Serum
WT
WT
MMP-13 KO
MMP-13 KO
SDF-1α　(ng/mg)
SDF-1α　(ng/ml)
 Cont
 Day 1
 Day 3
 Day 7
 Cont
 Day 1
 Day 3
 Day 7
