# Supplementary material for: Host-derived MMP-13 exhibits a protective role in lung metastasis of melanoma cells by local endostatin production
Source: Br J Cancer. 2011 Oct 20;105(10):1615–24. doi: 10.1038/bjc.2011.431 (PMC3242531; doi:10.1038/bjc.2011.431)
Supplement: Supplementary Information [file bjc2011431x2.doc]

**Supplementary Methods**

**Enzyme-linked immunosorbent assay (ELISA) for SDF-1α**

Mouse lung samples in RIPA buffer containing protease inhibitor cocktail (Complete Mini, Roche, Indianapolis, IN) were homogenized by Potter-Elvehjem homogenizer on ice. Homogenates were centrifuged for 10 min at 12,000*g*, and then supernatants were carefully aspirated. Total protein concentrations of the supernatants were determined by Proteostain (Dojindo, Kumamoto, Japan). Serum samples were also obtained by collecting blood samples from the heart. The supernatants and serum samples were subjected to the Quantikine mouse CXCL12/SDF-1α ELISA kit (MCX120, R&D Systems). Results of SDF-1α were calculated as ng/mg protein and ng/ml for homogenate supernatants and serum samples, respectively.

**Supplementary Figure 1.** The expression of SDF-1α (CXCL12) and CXCR4 in the lung tissues and the levels of SDF-1α in the lung and serum samples of WT and MMP-13 KO mice after intravenous injection of B16BL6 melanoma cells. (A) The mRNA expression of SDF-1α and CXCR4 in the lungs of WT and MMP-13 KO mice. Mice were received intravenous injection of B16BL6 cells and lung tissues obtained on days 0 (Cont), 1, 3 and 7 after the injection were subjected to RT-PCR for SDF-1α and CXCR4 as described in MATERIALS AND METHODS. β-actin, a loading control. (B) The SDF-1α levels in the lung and serum samples. The homogenate supernatants of the lung tissues and serum samples obtained on days 0 (Cont), 1, 3 and 7 after the injection in WT and MMP-13 KO mice (n=5 per group) were subjected to the ELISA assay for SDF-1α. Bars, mean ± SD.
